# Development of tailored indigenous marine consortia for the degradation of naturally weathered polyethylene films

**DOI:** 10.1371/journal.pone.0183984

**Published:** 2017-08-25

**Authors:** Evdokia Syranidou, Katerina Karkanorachaki, Filippo Amorotti, Eftychia Repouskou, Kevin Kroll, Boris Kolvenbach, Philippe F-X Corvini, Fabio Fava, Nicolas Kalogerakis

**Affiliations:** 1 School of Environmental Engineering, Technical University of Crete, Chania, Greece; 2 Department of Civil, Chemical, Environmental and Materials Engineering (DICAM), University of Bologna, Bologna, Italy; 3 School of Mineral Resources Engineering, Technical University of Crete, Chania, Greece; 4 Institute for Ecopreneurship, School of Life Sciences, FHNW, Muttenz, Switzerland; University of Oklahoma, UNITED STATES

## Abstract

This study investigated the potential of bacterial-mediated polyethylene (PE) degradation in a two-phase microcosm experiment. During phase I, naturally weathered PE films were incubated for 6 months with the indigenous marine community alone as well as bioaugmented with strains able to grow in minimal medium with linear low-density polyethylene (LLDPE) as the sole carbon source. At the end of phase I the developed biofilm was harvested and re-inoculated with naturally weathered PE films. Bacteria from both treatments were able to establish an active population on the PE surfaces as the biofilm community developed in a time dependent way. Moreover, a convergence in the composition of these communities was observed towards an efficient PE degrading microbial network, comprising of indigenous species. In acclimated communities, genera affiliated with synthetic (PE) and natural (cellulose) polymer degraders as well as hydrocarbon degrading bacteria were enriched. The acclimated consortia (indigenous and bioaugmented) reduced more efficiently the weight of PE films in comparison to non-acclimated bacteria. The SEM images revealed a dense and compact biofilm layer and signs of bio-erosion on the surface of the films. Rheological results suggest that the polymers after microbial treatment had wider molecular mass distribution and a marginally smaller average molar mass suggesting biodegradation as opposed to abiotic degradation. Modifications on the surface chemistry were observed throughout phase II while the FTIR profiles of microbially treated films at month 6 were similar to the profiles of virgin PE. Taking into account the results, we can suggest that the tailored indigenous marine community represents an efficient consortium for degrading weathered PE plastics.

## Introduction

Plastics are synthetic organic polymers that are manufactured from petrochemicals and show various characteristics such as plasticity and high molecular mass. Due to their desirable properties their worldwide production has been significantly increased. Namely, the global production has increased from less than a million tons per year in 1940’s [1] to approximately 310 million tons per year in 2014 [2]. Although a fraction of post-consumer production is being recovered, the overall discarded plastic mass reached 25.2 million tons in 2012 [2]. In recent years, the production of bio-based polymers is increasing [3] whereby new bioplastics are produced from renewable resources or conventional plastics are combined with bio-based components to yield novel plastics [4].

There exists limited knowledge about the amount and distribution of plastic litter in the environment and especially in the marine environment. Recent studies have tried to estimate the abundance and distribution patterns of floating plastics in oceans since plastic pollution has gained much attention [5–7]. For example, 5.25 trillion plastic debris with an average weight of ~269000 tons may float at the oceans, while the largest percentage is accumulated in the North Hemisphere [5]. With respect to the Mediterranean Sea, the measured concentrations and loadings of floating plastics characterize it as a highly accumulated region next to the subtropical gyres [8].

Higher amounts of buoyant plastics were expected in accordance with the production volumes and this underestimation is mainly attributed to the ingestion by animals and sinking to the sea floor [9–12]. Once plastic debris enters the oceans, it may undergo various physical, chemical and biological mechanisms that lead to their fragmentation, sedimentation and migration [13,14]. Incorporation of plastic litter in marine trophic web as well as its negative effects on marine biota has been addressed [9,13,15]. Moreover, plastics especially polyethylene, contain organic xenobiotics and accumulate hydrophobic toxins from the surrounding environment [16]. Consequently, it has been suggested that plastic wastes should be characterized as hazardous in order plastic litter to be treated appropriately and thus their input volumes into nature is reduced [17].

Polyethylene (PE) is the most produced plastic and the decrease of its accumulation may significantly contribute towards a plastic-free wastes environment [18]. In Europe, the demand of polyethylene reaches approximately 30% of the plastic demand [2]. Degradation of this polymer can be biotic by exploiting the microorganisms combined with physical or chemical methods (abiotic mechanisms). In particular, the hypothetical degradation pathway of polyethylene requires the biofilm formation on the polymer surface and then scission and oxidation follow as a result of the synergy of abiotic and biotic mechanisms [19]. It is important to mention that the underlying mechanism responsible for biodegradation has not been elucidated as of yet. Few studies have investigated the microbial-mediated polyethylene degradation and demonstrated promising results until now and even fewer studies have exploited marine biota [19–21]. There is scarce information available concerning the kinetics of plastic mineralization in marine environment. The weight of immersed low density polyethylene pieces reduced by 1.9% while the weight of high density pieces was reduced by 1.6% annually [22].

Various classifications of plastics according to their size have been proposed in recent years [23]. The plastic pieces used in this study can be characterized as mesoplastics (2 mm- 2 cm), while smaller plastics are characterized as microplastics (<2 mm) and plastics with size bigger than 2 cm are characterized as macroplastics [24]. Naturally weathered polyethylene (PE) films were collected from beaches and used for the degradation experiments since it is among the most commonly observed plastics in the marine environment [12]. In view of the proposition to *think globally and act locally*, a two-phase microcosm biodegradation experiment was performed under simulated marine conditions in order to explore marine bacteria capable to degrade plastic debris. With the aim to search for an efficient, polyethylene-degrading community, the indigenous pelagic community alone and bioaugmented with strains competent to grow in medium with LLDPE (linear low density polyethylene) as the sole carbon source were investigated concerning their ability to colonize PE films, induce damages on the polymer’s surface and measurable weight loss. In the second phase the efficiency of the tailored biofilm consortia (indigenous and bioaugmented) was assessed in degrading naturally weathered PE films and was compared with the results from the first phase. The objective of this phase was to elucidate the potential ability of acclimated communities to mineralize weathered PE pieces under simulated marine conditions. The succession of biofilm community on PE surfaces was monitored in order to identify the successful colonizers.

## Materials and methods

### Samples collection and preparation

Naturally weathered PE plastics were collected from two coastal sites in Northern Crete; Agios Onoufrios (coordinates: 35.549128, 24.061855) and Kalathas (coordinates: 35.554538, 24.085120), both in Chania, Greece. Permission was given by Mr. Emmanuel Vegliris, Environment Dept, Region of Crete, Chania Office. The identification of the plastics was performed according to the polymers identification symbols scheme, a triangle of three "chasing arrows", which encloses a number giving the plastic type. The plastics pieces with the numbers 2 that corresponds to HDPE (high density polyethylene) and 4 that corresponds to LDPE (low density polyethylene) were collected while those with no clear identification were not used. Seawater was collected from Agios Onoufrios and filtered through a 200 μm mesh [25], in order to remove the zooplanktonic organisms and stored at 4°C.

**Fig 1:**
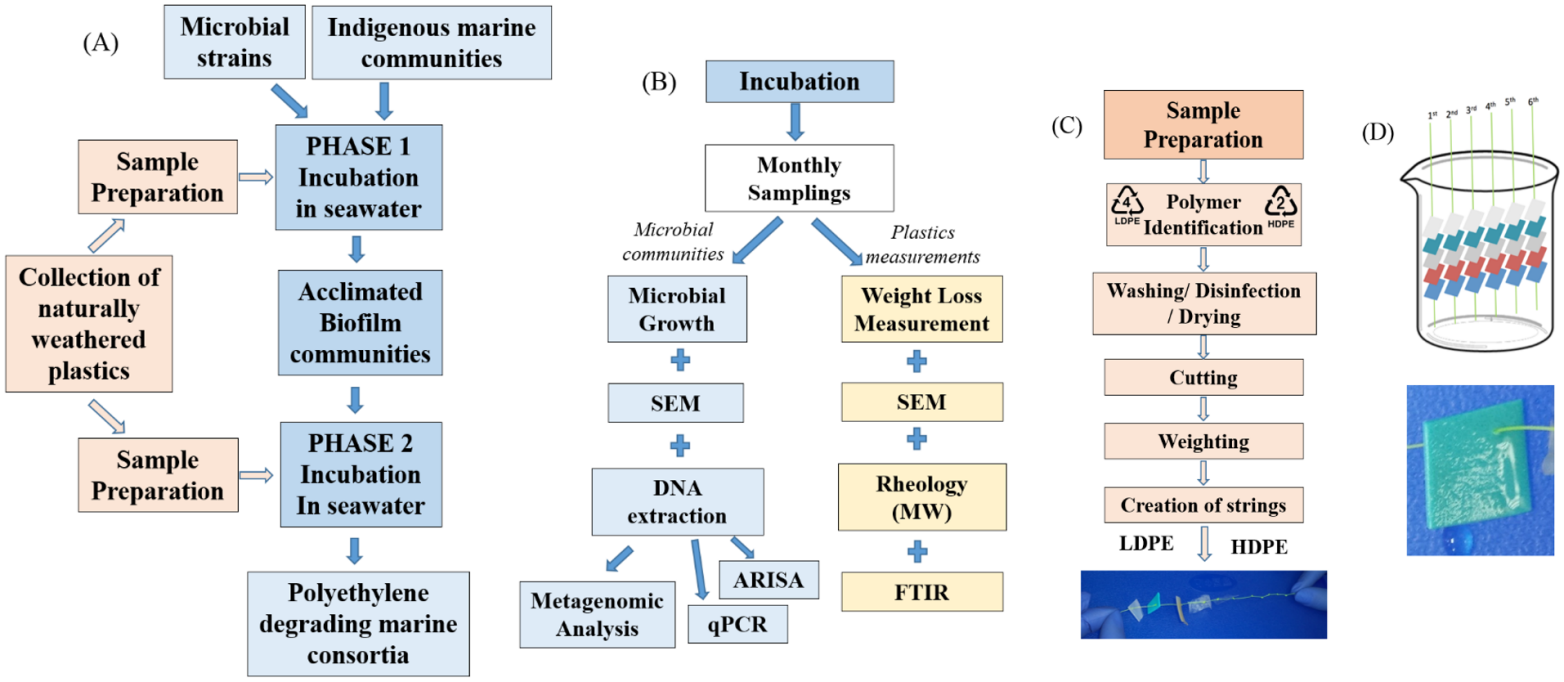
Experimental design. Schematic presentation of A) the experimental sequence; B) incubation and measurements of plastic properties and the microbial community, C) Sample preparation steps for setting up the strings with the plastic pieces, D) growth vessel with labelled strings with weathered plastic pieces of size 1 cm^2^ and biofilm development on plastic pieces.

The weathered plastics were cleaned using water and soap and were disinfected with 70% ethanol solution overnight. Next, they were dried at 50°C for 24h. Each plastic item was cut in many small pieces (approx. surface area: 1 cm^2^), weighed and strung from a fishing line. LDPE and HDPE pieces were put in the same fishing line. The weight of these flakes was approximately 15 mg. Each string of fishing line held 5 pieces of plastic and was assigned a number from 1 to 6, representing the month when sampling should occur. A combination of the string number with the position of each piece of plastic along the nylon string enabled the unique identification of each item (Fig 1).

### Bacterial strains

Bacterial isolates belonging to the genera *Lysinibacillus* and *Salinibacterium* were provided from Prof. Corvini’s lab (University of Applied Sciences and Arts, Switzerland). The strains (*Lysinibacillus* sp. and *Salinibacterium* sp.) originated from environmental samples collected either from plastic samples at Hooge Maey (Antwerp, Belgium) or surface water near Nordnes (Bergen, Norway) respectively. After successive enrichment cultures, they were found able to grow in mineral medium [26] supplemented with sterilized virgin linear LDPE (LLDPE) in powder form and 28 g L^-1^ NaCl to mimic seawater conditions.

Individual strains were incubated overnight in Standard I nutrient broth (7.8 g peptone from meat, 7.8 g peptone from casein, 2.8 g yeast extract, 5.6 g NaCl and 1 g glucose per 1000 mL distilled water) at 28°C under continuous shaking (120 rpm). When the OD_600_ reached 1.7 for *Lysinibacillus* sp. and 1.0 for *Salinibacterium* sp. the bacteria were harvested and washed three times with sterilized NaCl solution (8.5 g L^-1^).

### Experimental design

Biodegradation tests were performed in triplicates in pre-sterilized beakers (Fig 1). In phase I, the beakers contained 200 mL of the enriched filtered saline water (C:N:P ratio of 100:10:1) with the indigenous marine microbial community. Six strings with the sterile plastics flakes were added as the sole carbon source and one of the two treatments was bioaugmented by the LLDPE competent isolates (initial concentration: 1x10^8^ CFU mL^-1^). Namely, two different treatments were created: INDG and BIOG. The “indigenous” or “INDG” treatment corresponded to polyethylene pieces in seawater containing only the indigenous microorganisms whereas the “bioaugmented” or “BIOG” treatment corresponded to seawater with the indigenous population supplemented with *Lysinibacillus* sp. and *Salinibacterium* sp. Furthermore, two beakers containing 200 mL of sterile saline water and the pre-sterilized plastics serving as abiotic control were also monitored over time in order to determine the extent of abiotic degradation.

The beakers were incubated at 25°C on a stirring table at 120 rpm in darkness, and the whole experiment lasted 6 months. Sampling occurred at the end of each month by permanently removing one fishing line at a time from the microcosms (i.e., the three replicate microcosms were sampled at every time point, with each microcosm containing five plastic pieces, totalling 15 PE flakes). At the end of the biodegradation test of phase I, the biofilm communities that adhered to the plastic pieces were harvested by scraping the polymer surfaces with buffer solution and stored in glycerol solution at -80°C.

In phase II, the whole experiment was repeated using the acclimated biofilm communities (BIOG and INDG) as inoculants in order to quantify any potential enhancement in the degradation weathered PE samples by the acclimated communities.

### Development of a consortium

The acclimated biofilm communities were cultured in Standard I nutrient broth until the late log phase (the growth curves of each microbial community were previously performed by measuring the absorbance and cell numbers at same time intervals). They were further inoculated (initial concentration: 1x10^8^ CFU mL^-1^) in the beakers with enriched saline water and plastic pieces as the sole carbon source in the beginning of the biodegradation tests of phase II.

### Weight loss measurements

The measure of weight loss is a quick method to estimate the biodegradation of polymers assuming no abiotic processes take place that would cause weight reduction. Every plastic piece attached to the strings of the fishing line was washed off in order to remove the biofilm and then dried at 40°C for 3 days at each sampling point. Next, the flakes were weighted and the percentile weight loss from the original measurements (weathered plastic pieces used at the beginning of the bioaugmentation experiment) was determined in a 6-digit precision balance.

### Scanning electron microscopy (SEM)

Both non-treated and microbially treated plastic pieces were subjected to SEM analysis in order to observe potential bio-erosion on the surface of the polymers pieces as well as biofilm formation on the latter pieces. The plastic flakes were washed twice with 2% (v/v) aqueous sodium dodecyl sulphate solution for 30 minutes under mild shaking and then with distilled water. Immersion in 70% ethanol solution for 20 minutes was followed and then the samples were air-dried overnight. In order to observe the developed biofilm on the surface of the polyethylene pieces, the removed plastic pieces were washed with 0.1 M Phosphate Buffer (pH 7.2) for 20 minutes and fixed with 2% (v/v) formaldehyde for 2 hours under shaking. Subsequently, they were dehydrated by immersion into a graded series of ethanol solutions, namely, 25% ethanol for 30 minutes, 50% ethanol for 30 minutes, 75% ethanol for 30 minutes and 90% ethanol for 30 minutes. The samples were cut into small pieces, coated with gold and analysed under scanning electron microscope.

### Estimation of microbial growth of free and attached cells

During the biodegradation test of phase I, samples were taken from the water (aqueous phase) and from the attached biofilm by scraping the polymer’s surface at the end of the last sampling, while during phase II, samples were taken every month. Next, they were serially diluted and were spread on agar plates with Standard I medium. After 7 days of incubation at 25°C, the number of colonies was recorded and the CFUs per mL seawater for the free-living cells and per cm^2^ for the attached living cells were determined. The morphology of the colonies grown on the agar plates was compared to the morphology of the inoculated strains in order to verify their survival at the end of the experiment.

### Rheology measurements

The samples were shaped into discotic specimens at room temperature using a home-made vacuum mold along with a mechanical press (with applied pressure of 0.3 tons) and then placed on the rheometer at 165°C [27]. We used a stress-controlled rheometer MCR702 (Anton Paar, Austria). The specimen was placed between two stainless steel parallel plates of diameter 8 mm and gap about 0.7 mm. Temperature control was achieved by means of a hybrid temperature control system CTD180 which has intermediate characteristics between a Peltier cell and a convection oven. Nitrogen atmosphere was used for all the tests in order to reduce the risk of sample degradation. The measurement protocol consisted of three steps: (i) equilibration and stability of the samples was monitored by running frequency sweep tests in the same conditions at different times (starting from 20 min after loading, up to 1 hour) and checking for the overlap of the dynamic moduli (ii) dynamic strain sweeps at 100 rad/s and varying strain amplitude from 0.1% to 10% were carried out in order to detect the limits of linear viscoelastic response; an amplitude of 5% was found appropriate for all tests. (iii) dynamic frequency sweeps in the range from 300 to 0.1 rad/s were performed in order to probe the linear viscoelastic response. The interpretation of the movement of the intersection point is shown in S1 Fig.

### FTIR

Attenuated total reflectance—Fourier transform infrared spectroscopy (FTIR) was performed for the detection of the functional groups on the surface of the samples. A Frontier FT-IR spectrometer (PerkinElmer, Waltham, Massachusetts, USA) was used and the spectra were obtained and processed using PerkinElmer’s Spectrum software. Scan resolution was set at 4cm^-1^ for absorbance values ranging between 4000cm^-1^ and 650cm^-1^. Background scans for the reflectance of the surrounding atmosphere were performed before each sample scan and the sample’s peak heights were obtained by performing a baseline correction, subtracting the background spectrum from the sample spectrums. The percentage of crystallinity was calculated based on method suggested by Zerbi et al. [28].

$$\%\text{ Crystallinity }=\;\left(1\;-\;\frac{1-\left(\frac{I_{a}}{1.233I_{b}}\right)}{1+\left(\frac{I_{a}}{I_{b}}\right)}\right)\;100\%$$

where *I*_*a*_ and *I*_*b*_ are the absorbance values determined from the bands at 730cm^-1^ and 720cm^-1^, respectively.

### DNA extraction and ARISA PCR

DNA was isolated from the collected seawater and biofilm on the surface of at least three polymer pieces belonged to the same replicate of each treatment, pooled and eluted in Tris-EDTA (TE) buffer. During phase I, samples were taken only at the end of the experiment while on phase II samples were collected to monitor the succession of the adhered bacterial community. DNA extraction was performed according to the CTAB (Hexadecyltrimethylammonium bromide) protocol for the extraction of bacterial genomic DNA [29].

ARISA (Automated rRNA intergenic spacer analysis) PCR was performed in order to estimate the bacterial diversity among the different samples. The primers ITSF (5′-GTCGTAACAAGGTAGCCGTA-3′) and ITSReub (5′-GCCAAGGCATCCACC-3′) were used for the amplification of the ITS1 region in the rRNA operon plus ca. 282 bases of the 16S and 23S rRNA [29]. A mixture of 0.2 mM of each of the four deoxynucleoside triphosphates, 2 mM MgCl2, 0.2μM each of the forward and reverse primers and 1 U of High Fidelity Platinum Taq DNA polymerase per 25μL was used to perform the PCR. The cycling conditions of the PCR were: one denaturation phase at 94°C for 3 min, followed by 30 phases at 94°C for 45 s, 56°C for 45 s, 72°C for 2 min, and a final extension at 72°C for 7 min.

The gel-dye mix, marker, PCR products and ladder were loaded to the DNA chip according to the manufacture’s protocol (Agilent DNA 1000 Assay Protocol), next the chip was inserted to the Agilent 2100 Bioanalyzer (Agilent Technologies, Diegem, Belgium) and the chip run was executed.

### Metagenomic analysis based on 16S rDNA

The DNA concentration was determined using the Quantifluor dsDNA assay (Promega Corporation, USA). The concentration of the amplicons were measured and adjust to an equimolar amount of 4nM before sequencing. Next generation sequencing of 16S rDNA genes amplified from DNA extractions were performed according to Illumina’s application note (part # 15044223, Illumina, San Diego, USA). Primers for sequencing were 515F (5′-GTG CCA GCM GCC GCG GTA A-3′) and 806R (5′-GGA CTA CHV GGG TWT CTA AT-3′). PCR steps were performed using the KAPA HiFi HotStart kit (Kapa Biosystems, Wilmington, USA). The thermophaser program was the following: 95°C for 3 minutes, followed by 25 phases of 95°C for 30 seconds, 55°C for 30 seconds and 72°C for 30 seconds, respectively, with a final elongation step at 72°C for 5 minutes. The completed DNA libraries were run on the MiSeq Illumina, using a MiSeq Reagent Kit v3 (600-phase). The sequences were deposited in BioProject (PRJNA378706), the Submission ID is SUB2440072.

### Quantification of the *alkB* gene

The abundance of the *alkB* gene within acclimated biofilm communities was monitored via real-time PCR using a StepOne Plus System (Applied Biosystems Inc., Foster City, CA, USA). The primer pair used for quantification was *alkB*-f (5′- AAYACIGCICAYGARCTIGGICAYAA -3′) and *alkB*-r (5′ -GCRTGRTGRTCIGARTGICGYTG-3′) [30] while the expected amplicon size was in the range of 550 bp. qPCR master mix and conditions are performed as previously described [31]. All samples and standards were amplified in triplicates. For the standard curve, six-fold serial dilution of the *alkB* gene isolated from E8 consortium [32] was performed. The amplification efficiency and coefficient (r^2^) was 105% and 0.98 respectively. Melting-curve and a 1.5% agarose gel were used for assuring the specificity of the products.

### Data analysis

Statistical analysis was carried out with the automatic R software package [33]. Two way ANOVA was applied to the data following normal distribution in order to evaluate the effect of month or treatment to the different studied variables.

The analysis of ARISA fragment was performed with the Bioanalyzer software. Only peaks with sizes ranging between 100 and 1500 bp and a minimum peak height of 150 fluorescence units were considered for further analysis. The binning of ARISA fragments was performed according to Ramette [34], the automatic R [33] binning script was applied to replicates of the same treatment in order to find the window size (WS) and the shift value (Sh), and a WS of 1 bp was selected for the OTU binning algorithm for ARISA profiles for the planktonic and the attached cells. Next, the analysis of the OTU table was performed by Primer6 software and was analysed using the Bray–Curtis similarity method. A non-metric multidimensional scaling (nMDS) plot was used to describe the root community structure while the degree of similarity was explored with the permutation-based hypothesis statistical test ANOSIM. The Shannon–Wiener diversity index among the PE communities was calculated [35].

Next generation sequencing data analysis was performed on fastq files. After assembly of the paired-end reads, adapter trimming with a threshold of 0.9 and keeping amplicons with a length 605bp using PANDAseq version 2.8 [36], data were analyzed using the QIIME package, version 1.6.1 [37]. The default minimum quality threshold of 25 was used. The joined sequences were filtered and clustered *de novo* using the Greengenes database updated in May, 2013 (http://greengenes.lbl.gov) with a 97% identity threshold. Rarefied OTU tables were generated and all samples were subsampled to 4605 sequences per sample. Subsequently, 2d principal component analysis plots were prepared, using R [33] (package “phyloseq” [38]) while the ANOSIM statistical test was performed in QIIME. The linear discriminant analysis (LDA) effect size (LEfSe) [39] was performed to identify the biomarker species between the initial and acclimated biofilm communities.

## Results

### Microbial growth in microcosms

A microcosm experiment was conducted in two phases in order to evaluate the ability of two different marine consortia (the non-acclimated and acclimated marine community) to degrade weathered PE films (Fig 1D). Seawater was selected as the aqueous medium in order to simulate the pelagic zone and polyethylene was the only carbon source to allow the growth of only potential PE consumers.

Successful adaptation of the microorganisms on weathered PE surface would lead to the development of a viable community. During phase I, the BIOG consortium developed a visible biofilm on the weathered PE flakes after 4 months of incubation (Fig 1D) while biofilm formation was merely detected by naked eye on the PE samples in the indigenous treatment until the end of this phase. At that time, the biofilm populations of both treatments were harvested and cultured in order to verify that there were still active cells. Similar abundances were observed (bioaugmented: 3.7×10^4^ CFU cm^-2^; indigenous: 4×10^4^ CFU cm^-2^), indicating that the bacteria were able to establish population on the weathered plastic surface while using polyethylene as the sole carbon source. With respect to planktonic cells, the cell density was approximately 4 ×10^4^ CFU mL^-1^ in the bioaugmented beakers and 4.5×10^4^ CFU mL^-1^ in indigenous treatment. Taking into account that a population of 10^8^ cells mL^-1^ was added in the inoculated microcosms, a decrease in their abundance was noticed.

**Fig 2:**
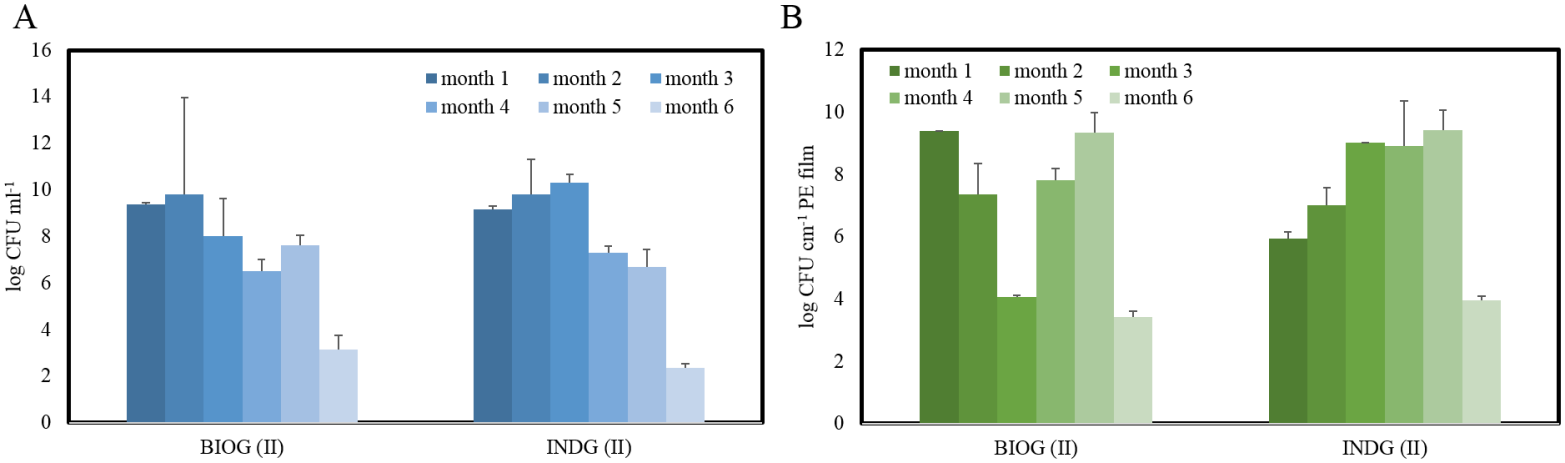
Cell densities. A) Abundances of free cells in the different treatments during phase II and B) abundances of the attached cells on the PE pieces during phase II.

Similar response of the BIOG planktonic cells was noticed during phase II of the experiment. More specifically, the abundances of BIOG free cells decreased from month 1 to month 6 (from 10^9^ CFU mL^-1^ to 10^3^ CFU mL^-1^ respectively) (Fig 2A). The concentration of the INDG cells increased until month 3, when they exhibited the highest concentration (10^10^ CFU mL^-1^). Afterwards, they decreased and 10^2^ CFU mL^-1^ were counted in the water samples of month 6. Two way ANOVA revealed significant effect of the factor month (F:122, p< 2×10^−16^) but no significant effect due to different treatment on the concentration of the cells.

It was demonstrated that both acclimated consortia were able to attach and colonize the weathered polymers’ surface faster in comparison to the non-acclimated ones. When they were incubated for two months, visible biofilm was detected on PE films in all treatments. Considering biodegradation of polyethylene as a slow procedure, the concentration of live biofilm cells was monitored to ensure metabolic activity during the experiment. As seen in Fig 2B, the bacteria were able to proliferate throughout the experiment. Two way ANOVA revealed significant effect of the month (F:6.9, p = 0.01) but no significant effect due to the different treatment on the concentration of the attached cells. However, the two different consortia displayed variations concerning the colonization efficiency and biofilm development. The BIOG cells demonstrated higher ability to attach to the weathered polymers’ surface compared to the indigenous community, since 10^9^ CFU cm^-2^ were enumerated from this treatment after one month incubation and 10^5^ CFU cm^-2^ from indigenous beakers. Interestingly, the bioaugmented biofilm population decreased until month 3, then it increased until month 5 and decreased again. At month 1 and 5, this population exhibited the maximum concentration of 10^9^ CFU cm^-2^. The indigenous biofilm community increased until month 3 to 10^9^ CFU cm^-2^, during month 4 and 5 remained stable and then it decreased to 10^4^ CFU cm^-2^.

### Weight reduction due to biodegradation

**Fig 3:**
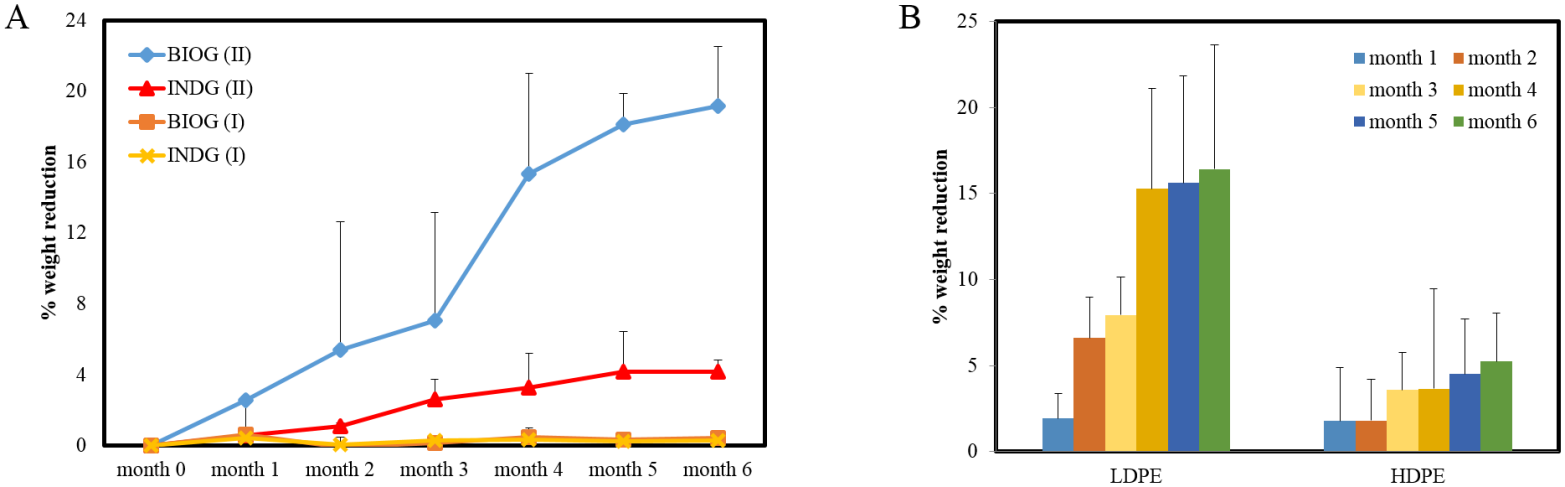
Weight reduction of PE films. A) Percentage of weight reduction by the different marine consortia (A: phase I, B: phase II); B) Percentage of weight reduction of the different polyethylene pieces during the phase II by both treatments.

In this experiment, the surface of all the plastic pieces was 1 cm^2^, since the weight reduction is proportional to the surface of polymer. The percentage of the weight reduction of weathered PE films owing to biodegradation is presented in Fig 3A. In phase I, after six months incubation, both non acclimated consortia decreased the PE films by a small fraction which corresponded to approximately 0.4% weight loss for bioaugmented treatment and 0.3% weight loss for the indigenous treatment.

A significant boost in the weight reduction was detected when the acclimated consortia were utilized. After one month incubation, the bioaugmented community decreased already 2.6% the weight of weathered PE samples while indigenous biofilm was responsible for 0.6% weight loss. At this phase, significant effects on weight of PE films were observed due to treatment (F:815, p< 2×10^−16^) and due to month (F:5, p = 0.026), while no interaction effect was detected between the two factors. The BIOG biofilm bacteria decreased the weight of weathered PE samples along time and after 6 months 19% of the mass was lost. A similar pattern was observed for the indigenous treatment, whereas this community reduced 4.2% of the PE weight after 6 months incubation. It is important to mention that no weight reduction was recorded when the sterile plastic pieces (abiotic control) were incubated with sterile saline water for a period of six months.

When the weight loss of weathered HDPE and LDPE flakes during phase II were compared, significant differences were revealed due to treatment (F: 74, p = 5×10^−5^) and due to month (F: 20, p = 1.2×10^−5^). Moreover, significant interaction effects (F: 9.8, p = 0.002) were noticed between the two factors. The two consortia reduced significantly the weight of LDPE pieces in comparison to the of HDPE pieces at month 2 and after month 4 (Fig 3B). More specifically, they decreased more than 15% the weight of LDPE flakes after month 4, while the weight loss of HDPE flakes reached 3.6% at month 4, 4.5% at month 5 and 5.2% at month 6.

### SEM analysis

**Fig 4:**
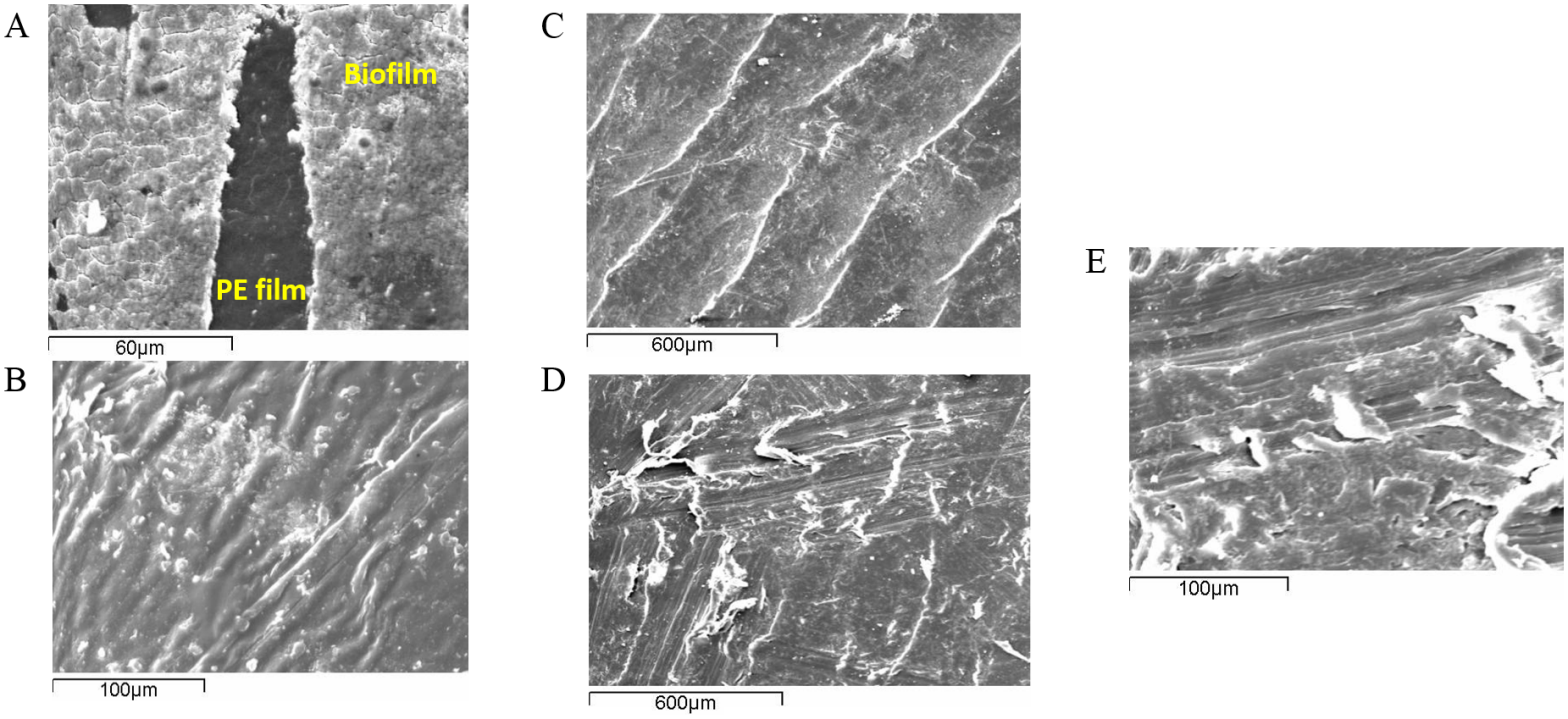
SEM analysis. SEM images of biofilm formed by the marine microbes on polyethylene: (A) at the end of first month, (B) at the end of the phase II and of the surface of the weathered pieces before (C) and after exposure to microbial treatment (D) & (E) at the end of phase II.

The adhesion of microorganisms on the plastic surface and the biofilm formation was elucidated with scanning electron microscopy (Fig 4A & 4B). At the end of phase I, the extent of colonization on the polymer surfaces was visually observed and a dense layer covering the plastic surfaces was observed. In order to monitor the microbial attachment, samples were taken every month during phase II. It was demonstrated that both consortia were able to adhere onto the PE pieces and established a population already from the first month, while no signs of attached bacteria/developed matrix were noticed on sterile PE films exposed to abiotic conditions (S2 Fig). After 6 months incubation, the plastic surfaces were also fully covered by a thick multi-layer matrix of material.

This technique was also used in order to verify any potential erosion on the surface due to microbial activity. The surface topography of non-treated samples and samples subjected to the consortia was compared and defects were detected on the treated samples. As seen in Fig 4C, the weathered PE films had a wavy appearance with some small cracks probably because they were exposed to UV radiation and temperature changes before they were collected. At the end of both phases, it seems that the waves disappeared and many fissures and small holes were created on the PE surface (Fig 4D & 4E).

### Rheological behavior of weathered and microbial-treated samples

**Fig 5:**
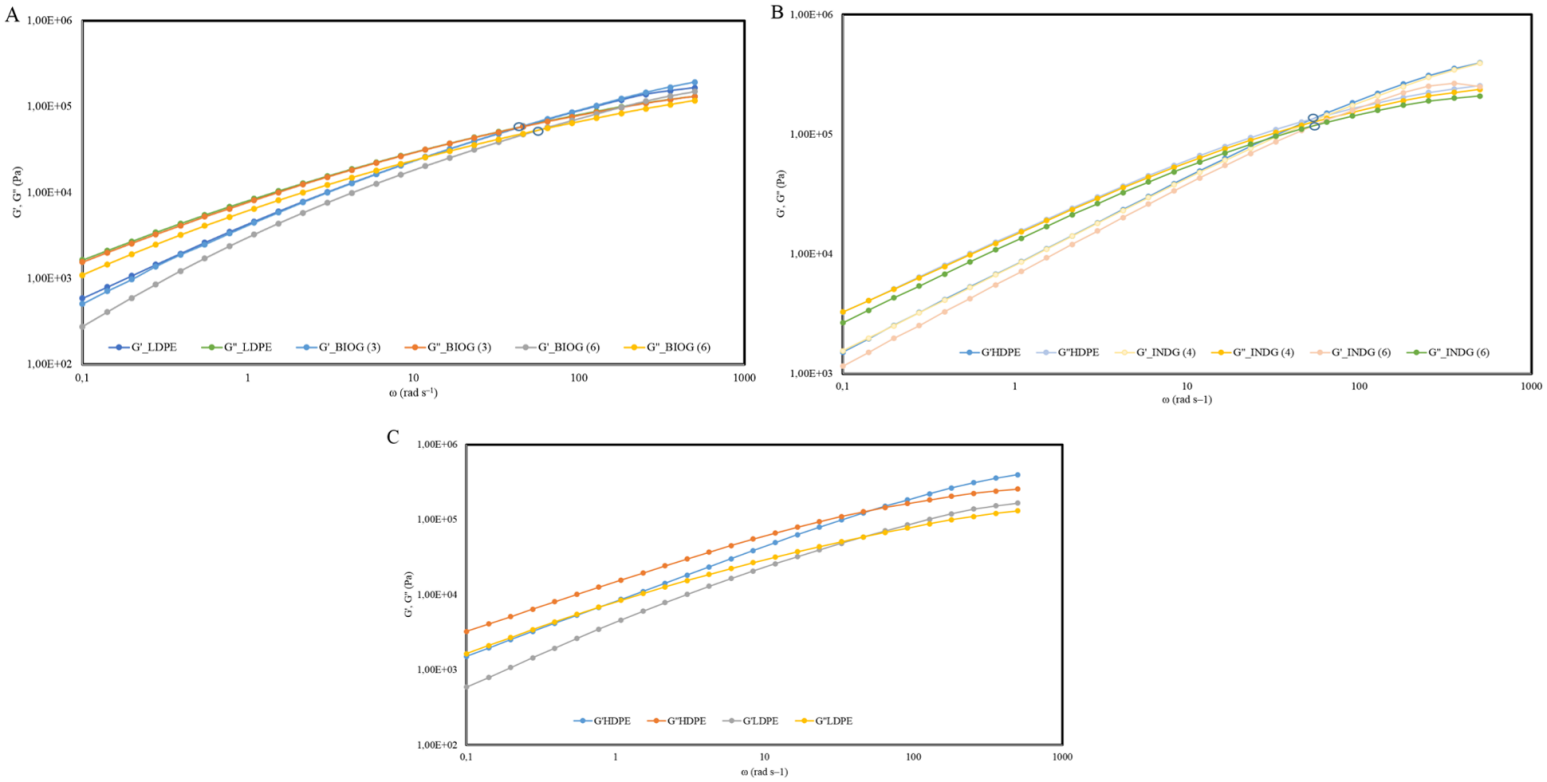
Rheological analysis. A) Master curves of storage (G΄) and loss (G΄΄) moduli as a function of frequency corresponding to the BIOG treatments of phase II, B) Master curves of storage (G΄) and loss (G΄΄) moduli as a function of frequency corresponding to the INDG treatments of phase II and C) Master curves of storage (G΄) and loss (G΄΄) moduli as a function of frequency corresponding to the weathered plastics.

With the aid of recent tube-model theories it is possible to determine the molecular weight distribution of polymers with rheological measurements [40]. For the present samples we restrict the discussion on the qualitative features of the measured viscoelastic spectra. The latter are represented in the form of plots of G΄and loss G΄΄ against angular frequency (ω) (Fig 5). In case of weathered LDPE treated with the BIOG consortia, it was demonstrated that the crossover point marking the onset of terminal regime (i.e., flow of the polymer) moved below (reduced modulus) within 6 months of incubation in comparison to the cross over point of the naturally weathered LDPE. A similar pattern was obtained for the weathered HDPE treated with the indigenous consortium. A shift to lower cross over point was noticed, although this shift occurred to a lesser extent. This is consistent with the lower weight reduction observed for this treatment, since 19% weight reduction was accomplished by the BIOG consortium in comparison to 4.2% accomplished by the indigenous community. These rheological measurements suggest that the molar mass distribution of the microbially-treated polymers has been likely broadened implying a shorter and less branched molecule. Moreover, the treated polymers have a marginally lower average molar mass, indicating a biodegradation effect as opposed to fragmentation due to abiotic stresses.

### FTIR

**Fig 6:**
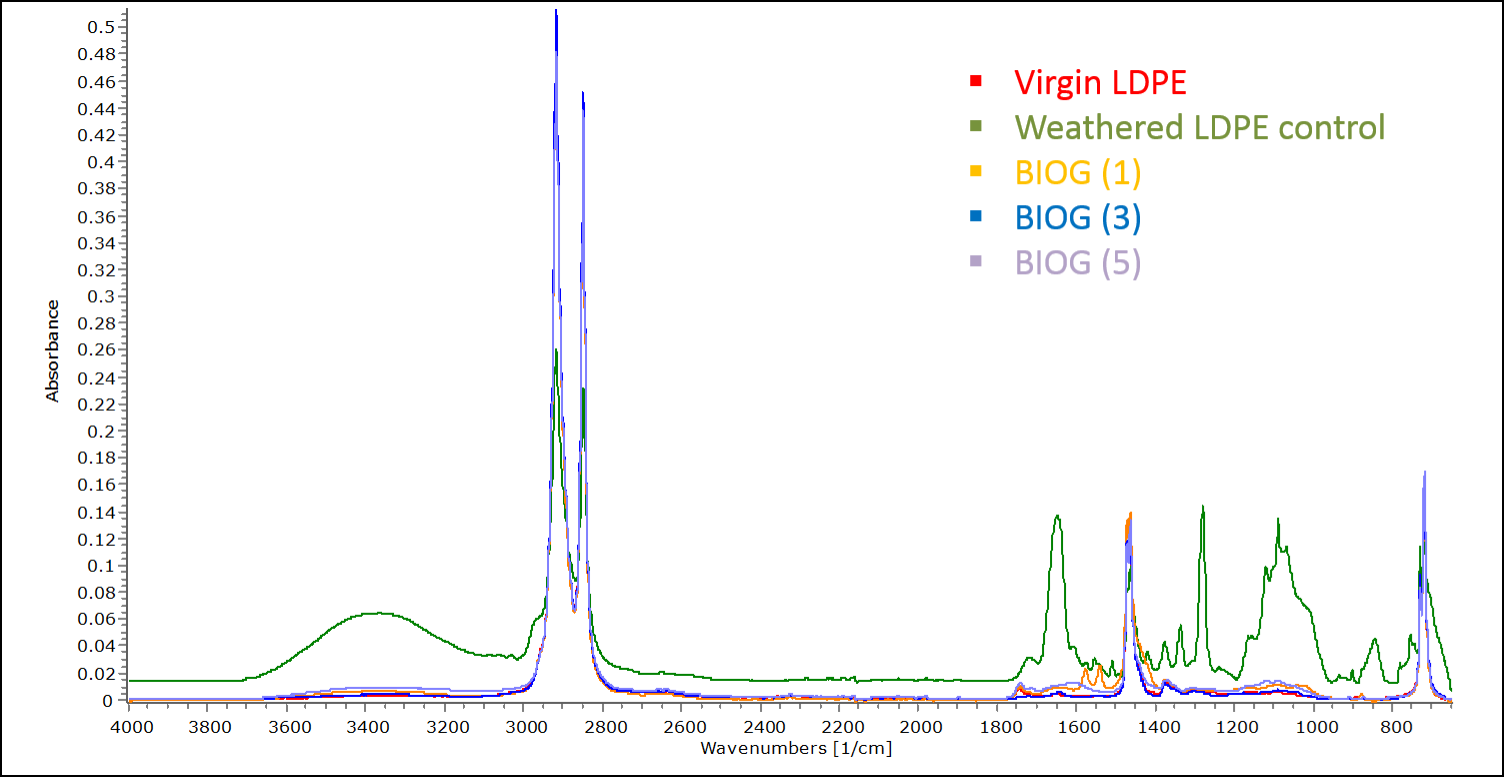
FTIR analysis. Spectra of virgin LDPE, naturally weathered LDPE, LDPE films exposed to BIOG microbial community for 1 month, LDPE films exposed to BIOG microbial community for 3 months and LDPE exposed to BIOG microbial community for 5 months.

Changes of the functional groups on surface of polyethylene films were monitored with FTIR spectroscopy (Fig 6 and S3 Fig) throughout the experimental period. The characteristic PE bands at 2,919, 2,850 cm^-1^ (-CH), 1,460 cm^−1^ and 1,470 cm^−1^ (-CH) and approximately 720–730 cm^−1^ (-CH) remained stable in all samples while various bands appeared at weathered or microbially treated films. Presence of–OH band which is in the range of 3600–3200 cm^−1^ can be detected in weathered LDPE films and microbially treated HDPE pieces. Similarly, the absorption peaks at approximately 1650 cm^−1^, 1550 cm^−1^, 1280 cm^−1^ and 1090 cm^−1^ represent double bonds and have appeared in the abovementioned samples. Interestingly, the spectra of microbially treated films at the end of experimental period are similar to the spectra of the virgin polymers. Moreover, crystallinity increased in LDPE weathered films in comparison to virgin ones and progressively decreased in the microbially treated films. With respect to HDPE pieces, crystallinity decreased in weathered films, increased in films at month 1 and again progressively decreased.

### PE-associated communities

**Fig 7:**
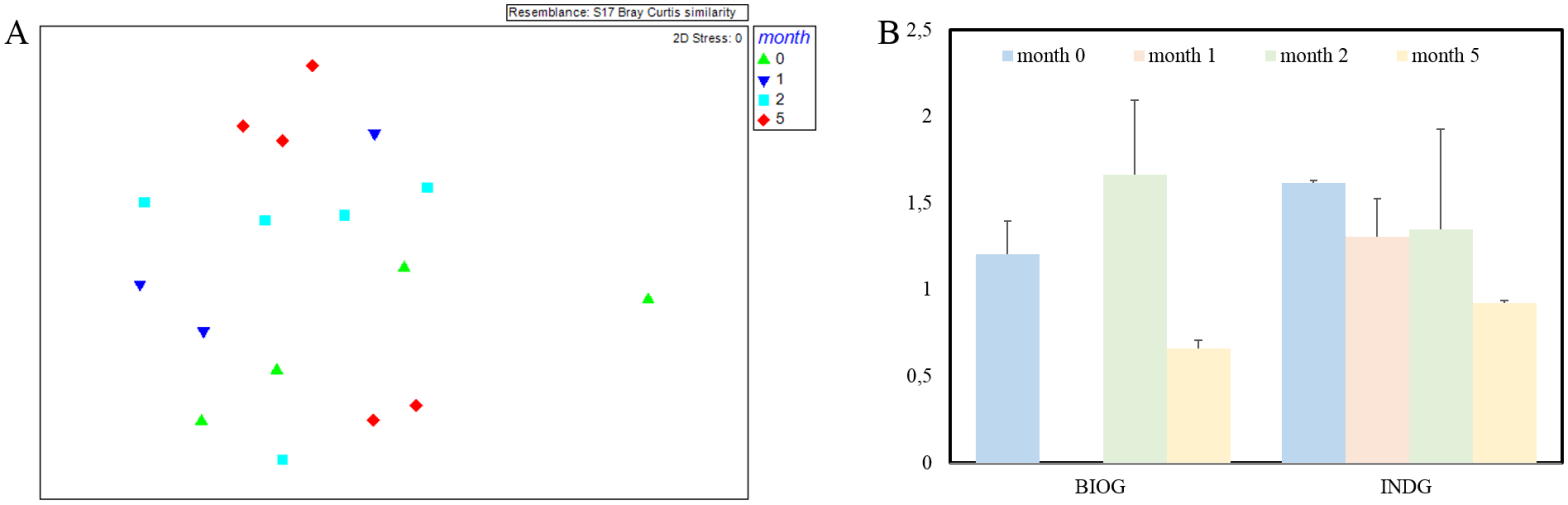
ARISA results. A) Non-metric multidimensional scaling (nMDS) ordination based on Bray–Curtis similarities from ARISA fingerprints of marine biofilm communities on the polyethylene pieces during the experiment (phase II), B) Shannon–Wiener diversity index among the different PE biofilm communities.

ARISA analysis was performed in the biofilm samples collected at the end of the phase I and during phase II, in order to monitor the succession of bacterial communities. The ARISA results corresponded to the BIOG microbial community (B) after one month incubation were excluded from statistical analysis due to low fluorescence signals. As seen in Fig 7A, the distribution of bacterial phylotypes changed with respect to time (ANOSIM R: 0.101, p< 0.05). It seems that the communities of the first two months were more similar to the initial while the biofilm structure of the month 5 was the most discrete. When the initial consortia were the selecting factor, no significant differences were detected among the communities adhered to the PE at various time intervals.

When comparing the diversities (Fig 7B), no significant effects were exhibited due to months of cultivation (F: 0.8, p = 0.39) or due to treatment (F: 0.57, p = 0.47). Moreover, no significant interaction effect was detected between the two factors. The indigenous assemblages tend to be less diverse along time while the diversity of bioaugmented community increased until the month 2 and then reduced.

**Fig 8:**
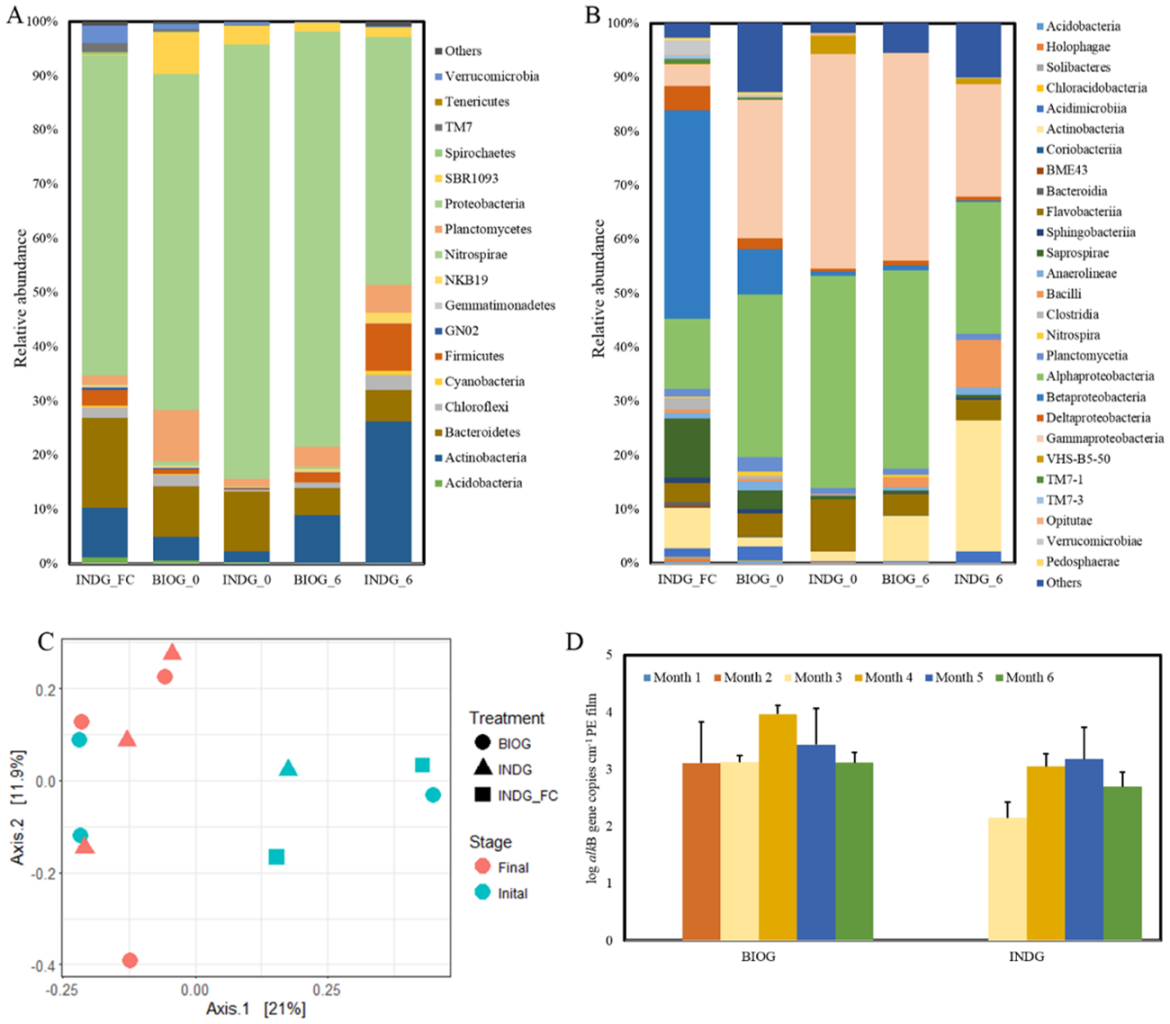
Microbial communities. Community composition of major (A) bacterial phyla and classes (B) of the biofilm communities, (C) PCoA plot of the PE adhered communities and (D) *alkB* gene copy numbers in biofilm communities.

Next to ARISA analysis, samples from the seawater used as the aqueous media as well as from the biofilm that was developed at the end of the two phases were sequenced with next generation sequencing techniques (16S rRNA gene sequencing using the MiSeq platform). The analysis of the community composition revealed that the phylum Proteobacteria dominates the planktonic and biofilm communities (Fig 8A), since it accounts for more than 50% of the read numbers of every community tested. Phyla Bacteroidetes and Actinobacteria were also abundant. Moreover, the relative abundance of p. Bacteroidetes was higher in samples collected from the water column and from the biofilm samples of phase I over the p. Actinobacteria while the opposite occurs in biofilm samples collected at the end of phase II. With respect to classes, Betaproteobacteria (36%) were most abundant in the planktonic assemblages while Alphaproteobacteria (~30%) and Gamma-proteobacteria (~28%) dominate most of the biofilm samples (Fig 8B).

The PCoA analysis using the unweighted UniFrac distance similarity metrics revealed that the factor group does not have a significant effect on the community structures (p> 0.05) (Fig 8C), suggesting that there are not significant differences between the bioaugmented and indigenous marine community compositions. Moreover, the inoculated strains were not detected in the biofilm communities harvested already at the end of the phase I, indicating that they could not dominate over the indigenous marine species or even survive. Based on these results, we infer that both acclimated biofilm communities are comprised of indigenous marine species. Moreover, the factor month significantly differentiates the bacterial assemblages (ANOSIM R:0.3, p< 0.05).

The abundance of *alkB* gene in acclimated adhered communities was monitored during phase II (Fig 8D). As revealed by two-way ANOVA, significant effect on the abundance of this gene was detected due to treatment (F: 21, p = 3×10^−5^) as well as due to month (F: 78, p = 6×10^−12^) while no interactive effects were observed. It seems that *alkB* exhibits higher concentration in bioaugmented communities in comparison to the indigenous ones. The gene was not detected in any community at the end of first month, while this was also the case for indigenous communities at the end of the second month. The highest abundance of *alkB* was measured at month 4 and 5 in bioaugmented and indigenous communities respectively.

**Fig 9:**
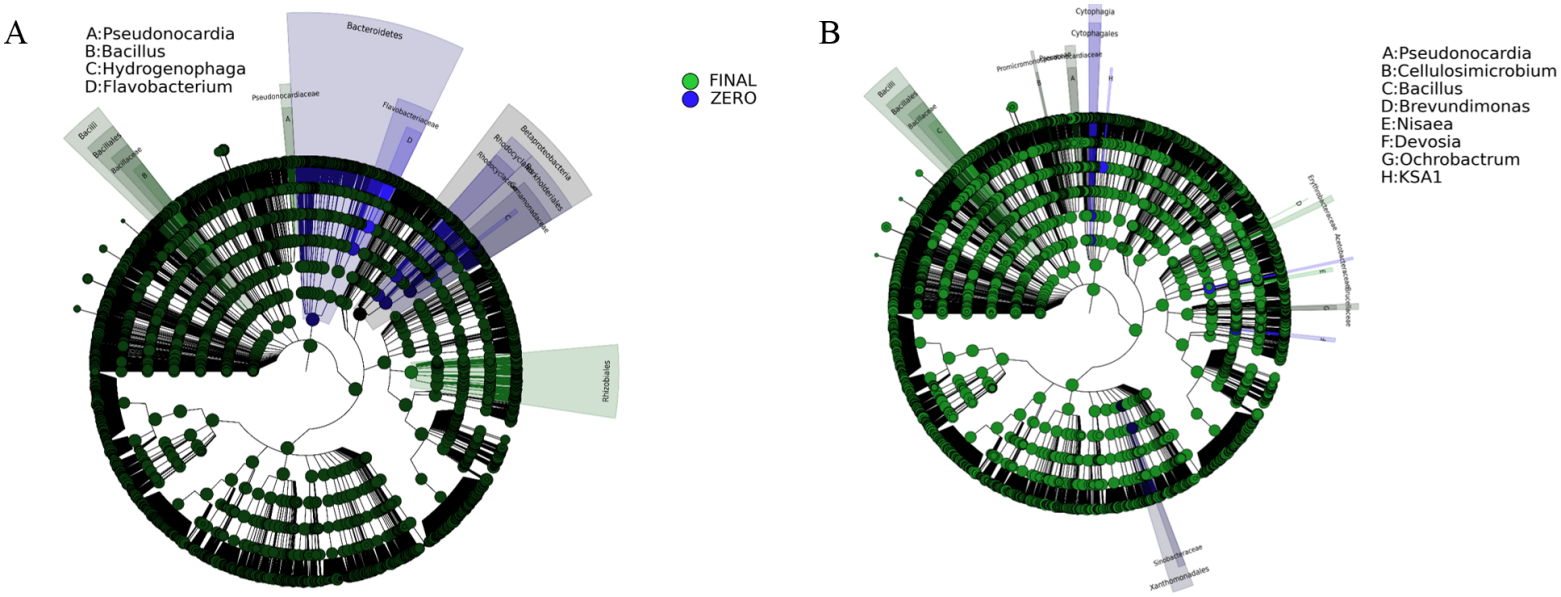
Biomarkers. Biofilm biomarkers of the initial consortium and the final developed communities. A) LEfSe was used to validate the statistical significance and the effect size of the differential abundances of taxa of INDG community (Kruskal-Wallis and Wilcoxon rank-sum p<0.05 and LDA score >4), B) LEfSe was used to validate the statistical significance and the effect size of the differential abundances of taxa of BIOG community (Kruskal-Wallis and Wilcoxon rank-sum p<0.05 and LDA score >3).

The Linear discriminant analysis (LDA) effect size (LEfSe) revealed the enriched OTUs in the acclimated biofilm communities. The abundance of bacteria affiliated with the genera *Pseudonocardia* and *Bacillus* was significantly increased in both well-developed PE associated assemblages (Fig 9A & 9B). The discriminant OTUs in final bioaugmented biofilm community were also assigned to the genera *Cellulosimicrobium* and *Ochrobactrum*. The abundances of taxa increased in the acclimated biofilm communities are presented in the S4 Fig.

## Discussion

Although polyethylene is considered non-biodegradable, studies have shown that weathering by exposure to photo-oxidation or thermal oxidation favor microbial attachment and degradation [41,42]. This process leads to carbonyl residues that can be used as carbon source by microorganisms. Besides thermal or light degradation, the abiotic pre-treatment also involves the mechanical and chemical impact of various factors on polymers [43].

In this context, naturally weathered PE pieces were collected from various beaches in Crete. A two-phase microcosm experiment was further conducted in order to evaluate the ability of different consortia to degrade polyethylene in the marine environment. During the phase I, the bioaugmented and indigenous marine communities were incubated with weathered PE pieces for 6 months. Since the biofilm formation is a prerequisite in polymer biodegradation process, only the viable biofilm cells were harvested and were further inoculated with PE pieces again for 6 months (phase II). It was demonstrated that biofilm cells possess structural and physiological characteristics that offer them high chances for adaption to LDPE surfaces in comparison to the planktonic cells [44].

The bacteria of both treatments were able to survive and thrive using weathered polyethylene as a sole carbon source. High numbers of biofilm cells were enumerated at the end of the first month, the population tended to increase until it reached a plateau and then decreased. The abundance of free cells decreased through time, but a population was maintained despite the carbon starvation. It can be hypothesized that the mature biofilms release dispersal cells to the water column during the experiment [45].

The polymer served not only as the sole carbon source but also as a substrate, underlying that the hydrophobicity of the planktonic consortia should have been enhanced by the carbon limitation. The efficient colonization of non-soluble surfaces is the first step in the polymer biodegradation and the excretion of extracellular enzymes follows [18]. Bacteria should overcome the hydrophobicity of the polymer with surfactant production or the strains with hydrophobic surfaces are the first colonizers [19]. Microorganisms adhere to the polymer and the breakdown of the big chain to smaller molecules initiates due to physical, chemical or enzymatic processes.

Both consortia developed a dense matrix of material, similar to those visualized of biofilms in literature [46] on the weathered polymer surfaces, as being visualized by the scanning electron microscopy. The formation of biofilm was was consistently observable on inoculated films in all the treatments independently of the type of polymer and the extent of weight loss. In general, HDPE films are not very attractive substances to adhere to and most bacteria display dispersed patterns of colonization [42,47]. The biofilm development was extensive in the first 30 days of incubation and no significant changes were further detected. Rapid biofilm formation was also noticed on polyethylene plastic food bags submersed in seawater [48]. A visible layer was developed on these bags already after one week exposure and kept increasing throughout the experiment. In ocean, immersed or buoyant plastics are susceptible to biofouling that leads to sinking and degradation [22,49].

Plastic niche presents a floating habitat thus harbors a variety of microorganisms and few invertebrate taxa [50,51]. Moreover, the plastic residents are metabolically active and distinct from the surrounding planktonic microbiota, comprising members of *Bryozoa*, *Cyanobacteria*, *Alphaproteobacteria*, and *Bacteroidetes* [52]. In this experiment, the planktonic bacterial community was not significantly different from the biofilm communities developed on PE surfaces. Members of Proteobacteria dominate all the bacterial assemblages while members of *Alphaproteobacteria* and *Gammaproteobacteria* are the most abundant classes in the biofilm samples.

It is difficult to identify the pioneer species and their role in the colonization process since the community composition alters within the first day [53] or over a longer period of time [54]. In accordance, monitoring of the microbial succession on weathered PE surfaces revealed a time dependent community structure, with shifts towards less diverse communities over months. During phase II where the degradation is more prominent, the well-developed biofilm communities differ significantly from the initial consortia, implying that an efficient microbial network has been developed on PE surfaces. Whereas, no significant difference was detected between the biofilm compositions of both treatments, underlying a convergence of the PE associated communities. These results imply that the substrate (weathered PE films) is responsible for shaping the biofilm bacterial community structure.

Significant increase in the abundance of specific bacterial genera such as *Bacillus* in the mature biofilm was observed, that has previously been associated with PE degradation [19,20]. At the same time, species participating in hydrocarbon or natural polymers degradation have been enriched in the acclimated biofilm community. For example, the genus *Pseudonocardia* carries the gene responsible for encoding *AlkB*-rubredoxin fused proteins, which is one of the key enzymes in alkane degradation pathway in bacteria [55]. Interestingly, the concentration of genus *Cellulosimicrobium* comprising of hydrocarbon and cellulose degraders [56,57] was only increased in the acclimated bioaugmented assemblages, where the highest weight reduction was recorded. Moreover, these two genera exhibit higher abundances in the bioaugmented community in accordance with the higher concentration of *alkB* gene. More specifically, it appears that *alkB* harboring bacteria are significantly stimulated within the bioaugmented biofilm population. The *alkB* gene encodes the alkane 1-monooxygenase and is considered one of the key participants in polyethylene degradation [19,58].

Since successful colonization and population establishment does not verify polymer degradation [59], weight reduction, surface images, monitoring of chemical changes on the surface and changes in the molecular weight were obtained in order to evaluate the degradation accomplished by the consortia. The extent of weight reduction varied and depended on several factors such as the type of polymer and the degree of acclimatization of the microorganisms. For example, the consortia reduced more efficiently the mass of weathered LDPE films in comparison to weathered HDPE films, since more than 15% weight reduction was observed for the LDPE and 5% for the HDPE. In a similar experiment, more than 20% weight loss in starch-blended and approximately 15% weight loss in thermally treated LDPE films was accomplished by marine bacteria while only 7–9% weight loss was exhibited for the thermally treated HDPE films [21]. LDPE is a more branched polymer in comparison to HDPE, the intermolecular forces are weaker, the tensile strength as well as density are lower and hence, it is more susceptible to degradation [20].

Higher weight reduction was demonstrated in phase II when acclimated consortia were exploited. In particular, the bioaugmented community decreased 19% the PE mass at the end of phase II and 0.4% after the first 6 months of incubation in the phase I. Similarly, 4.4% and 0.3% mass loss was accomplished by the acclimated and non-acclimated indigenous marine community respectively. These results imply that the assimilation of weathered plastics by a previously exposed to them community overcomes the fragmentation to microplastics since HDPE films were found resistant to abiotic fragmentation when immersed in seawater after 6-months incubation [60]. Various results with respect to weight reduction have been reported until now [61–63]. Marine strains, affiliated to *Arthrobacter* sp. and *Pseudomonas* sp., were able to reduce 12% and 15% the HDPE respectively after 30 days incubation [64].

Microbial activity induces changes on the surface chemistry that can be elucidated with FTIR spectroscopy. In detail, a decrease or increase in the concentration of functional groups serve as indication of biological activity which is further enhanced in oxidised substrates [19]. Modification on the intensity of bands of HDPE films subjected to microbial activity was demonstrated [63]. In this experiment, several bands were detected on the surface of weathered plastic films in comparison to virgin ones. Interestingly, these bands were depleted and the chemical structure of the pieces at the end of phase II was similar to the profile of virgin polymers. We believe that microorganisms consumed the weathered part of the PE film and then as they reached the virgin molecules which are much more resistant to biodegradation their concentration decreased. Besides the presence of functional groups on the surface, crystallinity is an important parameter in monitoring PE biodegradation. In general, abiotic parameters increase crystallinity by degrading the amorphous regions [65] while similar effects have also been reported in case of biodegradation [20]. A progressive decrease in crystallinity has been observed in phase II, in accordance with other studies [21,66]. Once microorganisms consume completely the amorphous regions of the polymer, they start to degrade the smaller crystals thus increasing the proportion of larger crystals [19,20].

Alteration of the rheological properties and molecular mass distribution serve as an indicator of polymer biodegradation [20,43]. However, the effect of microbial activity on the molecular weight of polyethylene is a controversial issue since many researchers demonstrate no change of the molecular weight while others observe decrease or increase [19]. For example, Hadad et al. [67] reported that the soil thermophilic *Brevibaccillus borstelensis* strain 707 decreased 34% the molecular weight of photo-oxidized PE after 90 days incubation. Whereas incubation with various microorganisms did not have a significant effect on molar mass of PE films [41]. In our experiments, the rheological results demonstrate that a shorter molecule with wider molecular mass distribution was produced after microbial attack.

Changes on the surface topography of the weathered polymer films further support the biodegradation hypothesis. Progressive erosion on the plastic substances was observed during the experiment; the longer the incubation period was the more fissures and cracks appeared. Alteration of the initial film surface due to microbial growth has been elucidated by many studies [19,62,68], where grooves and pits were detected superficially after polymers were subjected to biodegradation.

Unravelling the underlying mechanisms of plastic colonization and subsequently the role of platisphere community in PE degradation will help towards successful remediation strategies.

## Conclusions

It was demonstrated that tailored indigenous marine communities comprising of polymer and hydrocarbon degrader species have the potential to degrade naturally weathered PE films in the marine environment before they are turned into microplastics. The bacterial populations were able to develop a dense biofilm on the weathered PE surfaces and induced alterations on the surface topography and chemistry and on rheological properties along with the weight decrease of the samples. At the end of the phase II, it appears that the developed consortium has depleted most of the weathered polymer as confirmed by FTIR spectra and the remaining PE film is lightly or not at all weathered as its FTIR spectrum is very similar to the virgin PE spectrum.

## Supporting information

S1 Fig Qualitative explanation of intersection movement. Correlation between the molar mass distribution (MMD) and the viscoelastic behavior of the polymer.
(TIF)

S2 Fig SEM analysis. SEM image of PE films exposed to abiotic treatment at the end of phase I.
(TIF)

S3 Fig FTIR spectra. A) Spectra of HPDE samples and B) spectra of virgin LDPE and HDPE films.
(TIF)

S4 Fig Biomarkers. Abundances of biomarkers (enriched OTUs) in acclimated biofilm communities (INDG: Kruskal-Wallis and Wilcoxon rank-sum p<0.05 and LDA score >4 & BIOG: Kruskal-Wallis and Wilcoxon rank-sum p<0.05 and LDA score >3).
(TIF)

## References

[1] Thompson RC, Swan SH, Moore CJ, vom Saal FS. Our plastic age. Philos Trans R Soc Lond B Biol Sci. 2009;364: 1973–1976. doi: 10.1098/rstb.2009.0054 19528049 10.1098/rstb.2009.0054 PMC2874019

[2] Plastics Europe. Plastics—the Facts 2014/2015. An Analysis of European Plas- tics Production, Demand and Waste Data [Internet]. Plastics 2015 Brussels, Belgium; 2015.

[3] Shah AA, Hasan F, Hameed A, Ahmed S. Biological degradation of plastics: A comprehensive review. Biotechnol Adv. 2008;26: 246–265. doi: 10.1016/j.biotechadv.2007.12.005 18337047 10.1016/j.biotechadv.2007.12.005

[4] Prieto A. To be, or not to be biodegradable??? that is the question for the bio-based plastics. Microb Biotechnol. 2016;9: 652–657. 27477765 10.1111/1751-7915.12393 PMC4993184

[5] Eriksen M, Lebreton LCM, Carson HS, Thiel M, Moore CJ, Borerro JC, et al Plastic Pollution in the World’s Oceans: More than 5 Trillion Plastic Pieces Weighing over 250,000 Tons Afloat at Sea. PLoS One. 2014;9: 1–15. doi: 10.1371/journal.pone.0111913 25494041 10.1371/journal.pone.0111913 PMC4262196

[6] Cozar A, Echevarria F, Gonzalez-Gordillo JI, Irigoien X, Ubeda B, Hernandez-Leon S, et al Plastic debris in the open ocean. Proc Natl Acad Sci. 2014;111: 10239–10244. doi: 10.1073/pnas.1314705111 24982135 10.1073/pnas.1314705111 PMC4104848

[7] Goldstein MC, Titmus AJ, Ford M. Scales of spatial heterogeneity of plastic marine debris in the northeast Pacific Ocean. PLoS One. 2013;8 doi: 10.1371/journal.pone.0080020 24278233 10.1371/journal.pone.0080020 PMC3835860

[8] Cózar A, Sanz-Martín M, Martí E, González-Gordillo Ignacio J, Ubeda B, Gálvez J, et al Plastic accumulation in the mediterranean sea. PLoS One. 2015;10: 1–12. doi: 10.1371/journal.pone.0121762 25831129 10.1371/journal.pone.0121762 PMC4382178

[9] Santos RG, Andrades R, Fardim LM, Martins AS. Marine debris ingestion and Thayer’s law—The importance of plastic color. Environ Pollut. Elsevier Ltd; 2016;214: 585–588. doi: 10.1016/j.envpol.2016.04.024 27131818 10.1016/j.envpol.2016.04.024

[10] Tubau X, Canals M, Lastras G, Rayo X, Rivera J, Amblas D. Marine litter on the floor of deep submarine canyons of the Northwestern Mediterranean Sea: The role of hydrodynamic processes. Prog Oceanogr. Elsevier Ltd; 2015;134: 379–403. doi: 10.1016/j.pocean.2015.03.013

[11] Kowalski N, Reichardt AM, Waniek JJ. Sinking rates of microplastics and potential implications of their alteration by physical, biological, and chemical factors. Mar Pollut Bull. Elsevier Ltd; 2016;109: 310–319. doi: 10.1016/j.marpolbul.2016.05.064 27297594 10.1016/j.marpolbul.2016.05.064

[12] Andrady AL. Microplastics in the marine environment. Mar Pollut Bull. Elsevier Ltd; 2011;62: 1596–1605. doi: 10.1016/j.marpolbul.2011.05.030 21742351 10.1016/j.marpolbul.2011.05.030

[13] Wright SL, Thompson RC, Galloway TS. The physical impacts of microplastics on marine organisms: A review. Environ Pollut. Elsevier Ltd; 2013;178: 483–492. doi: 10.1016/j.envpol.2013.02.031 23545014 10.1016/j.envpol.2013.02.031

[14] Wang J, Tan Z, Peng J, Qiu Q, Li M. The behaviors of microplastics in the marine environment. Mar Environ Res. Elsevier Ltd; 2016;113: 7–17. doi: 10.1016/j.marenvres.2015.10.014 26559150 10.1016/j.marenvres.2015.10.014

[15] Cole M, Lindeque P, Halsband C, Galloway TS. Microplastics as contaminants in the marine environment: A review. Mar Pollut Bull. Elsevier Ltd; 2011;62: 2588–2597. doi: 10.1016/j.marpolbul.2011.09.025 22001295 10.1016/j.marpolbul.2011.09.025

[16] Teuten EL, Saquing JM, Knappe DRU, Barlaz MA, Jonsson S, Björn A, et al Transport and release of chemicals from plastics to the environment and to wildlife. Philos Trans R Soc Lond B Biol Sci. 2009;364: 2027–45. doi: 10.1098/rstb.2008.0284 19528054 10.1098/rstb.2008.0284 PMC2873017

[17] Rochman CM, Browne MA, Halpern BS, Hentschel BT, Hoh E, Karapanagioti HK, et al Policy: Classify plastic waste as hazardous. Nature. 2013;494: 169–71. doi: 10.1038/494169a 23407523 10.1038/494169a

[18] Sivan A. New perspectives in plastic biodegradation. Curr Opin Biotechnol. Elsevier Ltd; 2011;22: 422–6. doi: 10.1016/j.copbio.2011.01.013 21356588 10.1016/j.copbio.2011.01.013

[19] Restrepo-Flórez J-M, Bassi A, Thompson MR. Microbial degradation and deterioration of polyethylene—A review. Int Biodeterior Biodegradation. Elsevier Ltd; 2014;88: 83–90. doi: 10.1016/j.ibiod.2013.12.014

[20] Kumar Sen S, Raut S. Microbial degradation of low density polyethylene (LDPE): A review. J Environ Chem Eng. Elsevier B.V.; 2015;3: 462–473. doi: 10.1016/j.jece.2015.01.003

[21] Sudhakar M, Doble M, Murthy PS, Venkatesan R. Marine microbe-mediated biodegradation of low- and high-density polyethylenes. Int Biodeterior Biodegradation. 2008;61: 203–213. doi: 10.1016/j.ibiod.2007.07.011

[22] Artham T, Sudhakar M, Venkatesan R, Madhavan Nair C, Murty KVGK, Doble M. Biofouling and stability of synthetic polymers in sea water. Int Biodeterior Biodegrad. Elsevier Ltd; 2009;63: 884–890. doi: 10.1016/j.ibiod.2009.03.003

[23] da Costa JP, Santos PSM, Duarte AC, Rocha-Santos T. (Nano)plastics in the environment—Sources, fates and effects. Sci Total Environ. Elsevier B.V.; 2016;566–567: 15–26. doi: 10.1016/j.scitotenv.2016.05.041 27213666 10.1016/j.scitotenv.2016.05.041

[24] Ryan PG, Moore CJ, van Franeker J a, Moloney CL. Monitoring the abundance of plastic debris in the marine environment. Philos Trans R Soc Lond B Biol Sci. 2009;364: 1999–2012. doi: 10.1098/rstb.2008.0207 19528052 10.1098/rstb.2008.0207 PMC2873010

[25] Hwang SJ, Heath RT. Zooplankton bacterivory at coastal and offshore sites of Lake Erie. J Plankt Res. 1999;Vol 21: 699–719. doi: 10.1093/plankt/21.4.699

[26] Stanier RY, Palleroni NJ, Doudoroff M. The Aerobic Pseudomonads: a Taxonomic Study. J Gen Microbial. 1966;43: 159–271. 10.1099/00221287-43-2-159 5963505

[27] Costanzo S, Scherz LF, Schweizer T, Kröger M, Floudas G, Schlüter AD, et al Rheology and packing of Dendronized polymers. Macromolecules. 2016;49: 7054–7068.

[28] Zerbi G, Gallino G, Del Fanti N, Baini L. Structural depth profiling in polyethylene films by multiple internal reflection infra-red spectroscopy. Polymer (Guildf). 1989;30: 2324–2327. doi: 10.1016/0032-3861(89)90269-3

[29] Cardinale M, Brusetti L, Quatrini P, Borin S, Puglia AM, Rizzi A, et al Comparison of Different Primer Sets for Use in Automated Ribosomal Intergenic Spacer Analysis of Complex Bacterial Communities. Appl Environ Microbiol. 2004;70: 6147–6156. doi: 10.1128/AEM.70.10.6147-6156.2004 15466561 10.1128/AEM.70.10.6147-6156.2004 PMC522057

[30] Perez-de-Mora A, Engel M, Schloter M. Abundance and Diversity of n-Alkane-Degrading Bacteria in a Forest Soil Co-Contaminated with Hydrocarbons and Metals: A Molecular Study on alkB Homologous Genes. Microb Ecol. 2011;62: 959–972. doi: 10.1007/s00248-011-9858-z 21567188 10.1007/s00248-011-9858-z

[31] Jin CE, Kim MN. Change of bacterial community in oil-polluted soil after enrichment cultivation with low-molecular-weight polyethylene. Int Biodeterior Biodegradation. Elsevier Ltd; 2017;118: 27–33. doi: 10.1016/j.ibiod.2017.01.020

[32] Antoniou E, Fodelianakis S, Korkakaki E, Kalogerakis N. Biosurfactant production from marine hydrocarbon-degrading consortia and pure bacterial strains using crude oil as carbon source. Front Microbiol. 2015;6: 1–14. 25904907 10.3389/fmicb.2015.00274 PMC4387541

[33] R Development Core Team. R: A Language and Environment for Statistical Computing. R Foundation for Statistical Computing R Foundation for Statistical Computing. Vienna, Austria; 2009.

[34] Ramette A. Quantitative community fingerprinting methods for estimating the abundance of operational taxonomic units in natural microbial communities. Appl Environ Microbiol. 2009;75: 2495–2505. doi: 10.1128/AEM.02409-08 19201961 10.1128/AEM.02409-08 PMC2675222

[35] Shannon CE, Weaver W. The Mathematical Theory of Communication. University of Illinois Press, editor. 2002.

[36] Masella AP, Bartram AK, Truszkowski JM, Brown DG, Neufeld JD. PANDAseq: paired-end assembler for illumina sequences. BMC Bioinformatics. 2012;13: 31 doi: 10.1186/1471-2105-13-31 22333067 10.1186/1471-2105-13-31 PMC3471323

[37] Caporaso JG, Kuczynski J, Stombaugh J, Bittinger K, Bushman FD, Costello EK, et al QIIME allows analysis of high-throughput community sequencing data. Nat Methods. 2010;7: 335–336. doi: 10.1038/nmeth.f.303 20383131 10.1038/nmeth.f.303 PMC3156573

[38] McMurdie PJ, Holmes S. Phyloseq: An R Package for Reproducible Interactive Analysis and Graphics of Microbiome Census Data. PLoS One. 2013;8 doi: 10.1371/journal.pone.0061217 23630581 10.1371/journal.pone.0061217 PMC3632530

[39] Segata N, Izard J, Waldron L, Gevers D, Miropolsky L, Garrett WSW, et al Metagenomic biomarker discovery and explanation. Genome Biol. 2011;12: R60 doi: 10.1186/gb-2011-12-6-r60 21702898 10.1186/gb-2011-12-6-r60 PMC3218848

[40] Dealy JM, Larson RG. Structure and Rheology of Molten Polymers, From Structure to Flow Behavior and Back Again [Internet]. NY: Carl Hanser Verlag GmbH & Co. KG; 2006 doi: 10.3139/9783446412811.fm

[41] Bonhomme S, Cuer A, Delort AM, Lemaire J, Sancelme M, Scott G. Environmental biodegradation of polyethylene. Polym Degrad Stab. 2003;81: 441–452. doi: 10.1016/S0141-3910(03)00129-0

[42] Fontanella S, Bonhomme S, Koutny M, Husarova L, Brusson JM, Courdavault JP, et al Comparison of the biodegradability of various polyethylene films containing pro-oxidant additives. Polym Degrad Stab. 2010;95: 1011–1021. doi: 10.1016/j.polymdegradstab.2010.03.009

[43] Lucas N, Bienaime C, Belloy C, Queneudec M, Silvestre F, Nava-Saucedo J-E. Polymer biodegradation: Mechanisms and estimation techniques—A review. Chemosphere. 2008;73: 429–442. doi: 10.1016/j.chemosphere.2008.06.064 18723204 10.1016/j.chemosphere.2008.06.064

[44] Tribedi P, Das Gupta A, Sil AK. Adaptation of Pseudomonas sp. AKS2 in biofilm on low-density polyethylene surface: an effective strategy for efficient survival and polymer degradation. Bioresour Bioprocess. 2015;2: 14 doi: 10.1186/s40643-015-0044-x

[45] McDougald D, Rice SA, Barraud N, Steinberg PD, Kjelleberg S. Should we stay or should we go: mechanisms and ecological consequences for biofilm dispersal. Nat Rev Microbiol. Nature Publishing Group; 2011;10: 39–50. doi: 10.1038/nrmicro2695 22120588 10.1038/nrmicro2695

[46] Tribedi P, Sil AK. Low-density polyethylene degradation by Pseudomonas sp. AKS2 biofilm. Environ Sci Pollut Res. 2013;20: 4146–4153. doi: 10.1007/s11356-012-1378-y 23242625 10.1007/s11356-012-1378-y

[47] Koutny M, Sancelme M, Dabin C, Pichon N, Delort AM, Lemaire J. Acquired biodegradability of polyethylenes containing pro-oxidant additives. Polym Degrad Stab. 2006;91: 1495–1503. doi: 10.1016/j.polymdegradstab.2005.10.007

[48] Lobelle D, Cunliffe M. Early microbial biofilm formation on marine plastic debris. Mar Pollut Bull. Elsevier Ltd; 2011;62: 197–200. doi: 10.1016/j.marpolbul.2010.10.013 21093883 10.1016/j.marpolbul.2010.10.013

[49] Fazey FMC, Ryan PG. Biofouling on buoyant marine plastics: An experimental study into the effect of size on surface longevity. Environ Pollut. Elsevier Ltd; 2016;210: 354–360. doi: 10.1016/j.envpol.2016.01.026 26803792 10.1016/j.envpol.2016.01.026

[50] Reisser J, Shaw J, Hallegraeff G, Proietti M, Barnes DKA, Thums M, et al Millimeter-sized marine plastics: A new pelagic habitat for microorganisms and invertebrates. PLoS One. 2014;9: 1–11. doi: 10.1371/journal.pone.0100289 24941218 10.1371/journal.pone.0100289 PMC4062529

[51] Zettler ER, Mincer TJ, Amaral-Zettler L A.. Life in the “Plastisphere”: Microbial communities on plastic marine debris. Environ Sci Technol. 2013;47: 7137–7146. doi: 10.1021/es401288x 23745679 10.1021/es401288x

[52] Bryant JA, Clemente TM, Viviani DA, Fong AA, Thomas KA, Kemp P, et al Diversity and Activity of Communities Inhabiting Plastic Debris in the North Pacific Gyre. mSystems. 2016;1: e00024–16. doi: 10.1128/mSystems.00024-16 27822538 10.1128/mSystems.00024-16 PMC5069773

[53] Lee JW, Nam JH, Kim YH, Lee KH, Lee DH. Bacterial communities in the initial stage of marine biofilm formation on artificial surfaces. J Microbiol. 2008;46: 174–182. doi: 10.1007/s12275-008-0032-3 18545967 10.1007/s12275-008-0032-3

[54] Harrison JP, Schratzberger M, Sapp M, Osborn AM. Rapid bacterial colonization of low-density polyethylene microplastics in coastal sediment microcosms. BMC Microbiol. 2014;14: 232 doi: 10.1186/s12866-014-0232-4 25245856 10.1186/s12866-014-0232-4 PMC4177575

[55] Nie Y, Chi C-Q, Fang H, Liang J-L, Lu S-L, Lai G-L, et al Diverse alkane hydroxylase genes in microorganisms and environments. Sci Rep. 2014;4: 4968 doi: 10.1038/srep04968 24829093 10.1038/srep04968 PMC4021335

[56] Nkem BM, Halimoon N, Yusoff FM, Johari WLW, Zakaria MP, Medipally SR, et al Isolation, identification and diesel-oil biodegradation capacities of indigenous hydrocarbon-degrading strains of Cellulosimicrobium cellulans and Acinetobacter baumannii from tarball at Terengganu beach, Malaysia. Mar Pollut Bull. Elsevier Ltd; 2016;107: 261–268. doi: 10.1016/j.marpolbul.2016.03.060 27085593 10.1016/j.marpolbul.2016.03.060

[57] Lo YC, Saratale GD, Chen WM, Der Bai M, Chang JS. Isolation of cellulose-hydrolytic bacteria and applications of the cellulolytic enzymes for cellulosic biohydrogen production. Enzyme Microb Technol. 2009;44: 417–425. doi: 10.1016/j.enzmictec.2009.03.002

[58] Pathak VM, Navneet. Review on the current status of polymer degradation: a microbial approach. Bioresour Bioprocess. Springer Berlin Heidelberg; 2017;4: 15 doi: 10.1186/s40643-017-0145-9

[59] Nauendorf A, Krause S, Bigalke NK, Gorb E V., Gorb SN, Haeckel M, et al Microbial colonization and degradation of polyethylene and biodegradable plastic bags in temperate fine-grained organic-rich marine sediments. Mar Pollut Bull. Elsevier Ltd; 2016;103: 168–178. doi: 10.1016/j.marpolbul.2015.12.024 26790603 10.1016/j.marpolbul.2015.12.024

[60] Kalogerakis N, Karkanorachaki K, Kalogerakis GC, Elisavet I, Gotsis AD, Partsinevelos P, et al Microplastics generation: Onset of fragmentation of polyethylene films in marine environment mesocosms. Front Mar Sci. 2017;4 doi: 10.3389/fmars.2017.00084

[61] Orr Gilan I, Hadar Y, Sivan A. Colonization, biofilm formation and biodegradation of polyethylene by a strain of Rhodococcus ruber. Appl Microbiol Biotechnol. 2004;65: 97–104. doi: 10.1007/s00253-004-1584-8 15221232 10.1007/s00253-004-1584-8

[62] Das MP, Kumar S. An approach to low-density polyethylene biodegradation by Bacillus amyloliquefaciens. 3 Biotech. 2015;5: 81–86. doi: 10.1007/s13205-014-0205-1 28324364 10.1007/s13205-014-0205-1 PMC4327746

[63] Kowalczyk A, Chyc M, Ryszka P, Latowski D. Achromobacter xylosoxidans as a new microorganism strain colonizing high-density polyethylene as a key step to its biodegradation. Environ Sci Pollut Res. 2016; 1–8. doi: 10.1007/s11356-016-6563-y 27072033 10.1007/s11356-016-6563-y PMC4884572

[64] Balasubramanian V, Natarajan K, Hemambika B, Ramesh N, Sumathi CS, Kottaimuthu R, et al High-density polyethylene (HDPE)-degrading potential bacteria from marine ecosystem of Gulf of Mannar, India. Lett Appl Microbiol. 2010;51: 205–211. doi: 10.1111/j.1472-765X.2010.02883.x 20586938 10.1111/j.1472-765X.2010.02883.x

[65] Roy PK, Titus S, Surekha P, Tulsi E, Deshmukh C, Rajagopal C. Degradation of abiotically aged LDPE films containing pro-oxidant by bacterial consortium. Polym Degrad Stab. 2008;93: 1917–1922. doi: 10.1016/j.polymdegradstab.2008.07.016

[66] Harshvardhan K, Jha B. Biodegradation of low-density polyethylene by marine bacteria from pelagic waters, Arabian Sea, India. Mar Pollut Bull. Elsevier Ltd; 2013;77: 100–106. doi: 10.1016/j.marpolbul.2013.10.025 24210946 10.1016/j.marpolbul.2013.10.025

[67] Hadad D, Geresh S, Sivan A. Biodegradation of polyethylene by the thermophilic bacterium Brevibacillus borstelensis. J Appl Microbiol. 2005;98: 1093–1100. doi: 10.1111/j.1365-2672.2005.02553.x 15836478 10.1111/j.1365-2672.2005.02553.x

[68] Raghul SS, Bhat SG, Chandrasekaran M, Francis V, Thachil ET. Biodegradation of polyvinyl alcohol-low linear density polyethylene-blended plastic film by consortium of marine benthic vibrios. Int J Environ Sci Technol. 2014;11: 1827–1834. doi: 10.1007/s13762-013-0335-8

