# Supplementary figures and images for: Development of tailored indigenous marine consortia for the degradation of naturally weathered polyethylene films
Source: PLoS One. 2017 Aug 25;12(8):e0183984. doi: 10.1371/journal.pone.0183984 (PMC5571942; doi:10.1371/journal.pone.0183984)

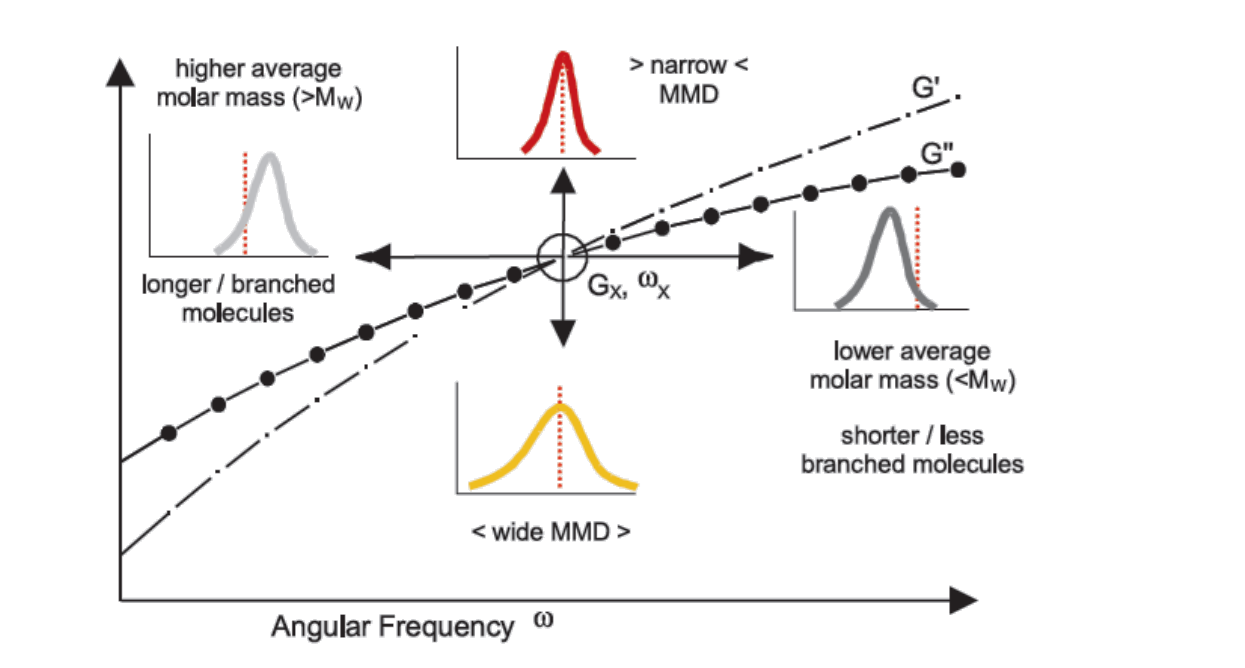

Supplement: S1 Fig — Correlation between the molar mass distribution (MMD) and the viscoelastic behavior of the polymer. (TIF) [file pone.0183984.s001.tif]

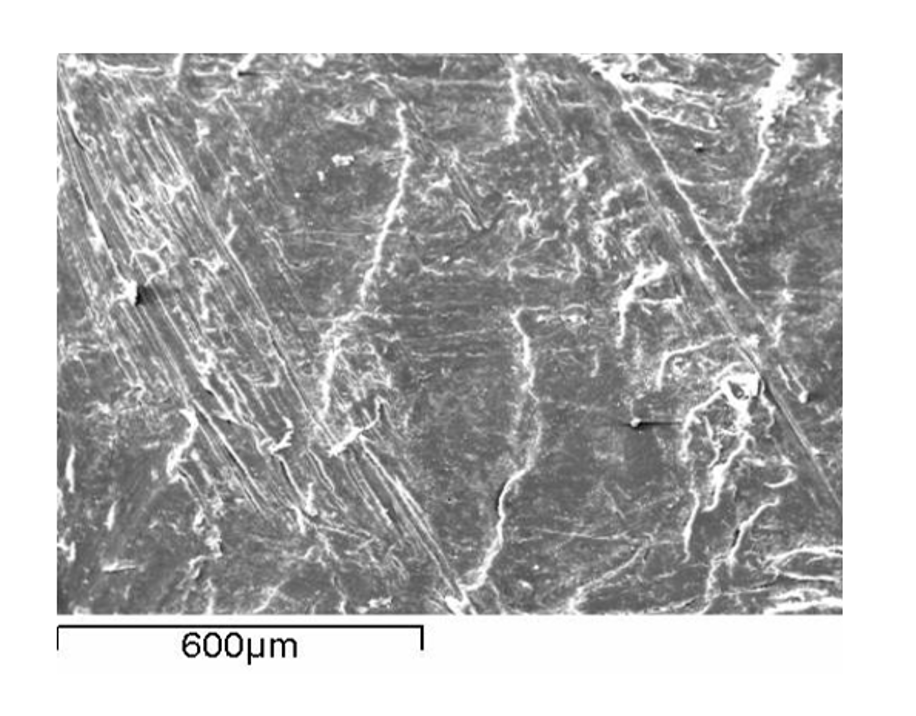

Supplement: S2 Fig — SEM image of PE films exposed to abiotic treatment at the end of phase I. (TIF) [file pone.0183984.s002.tif]

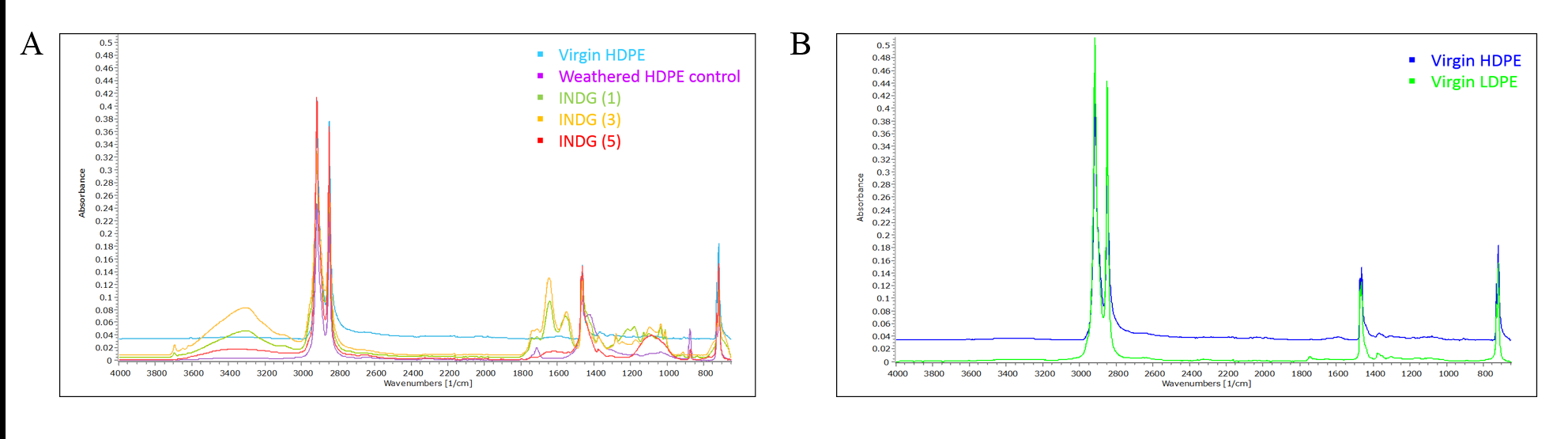

Supplement: S3 Fig — A) Spectra of HPDE samples and B) spectra of virgin LDPE and HDPE films. (TIF) [file pone.0183984.s003.tif]

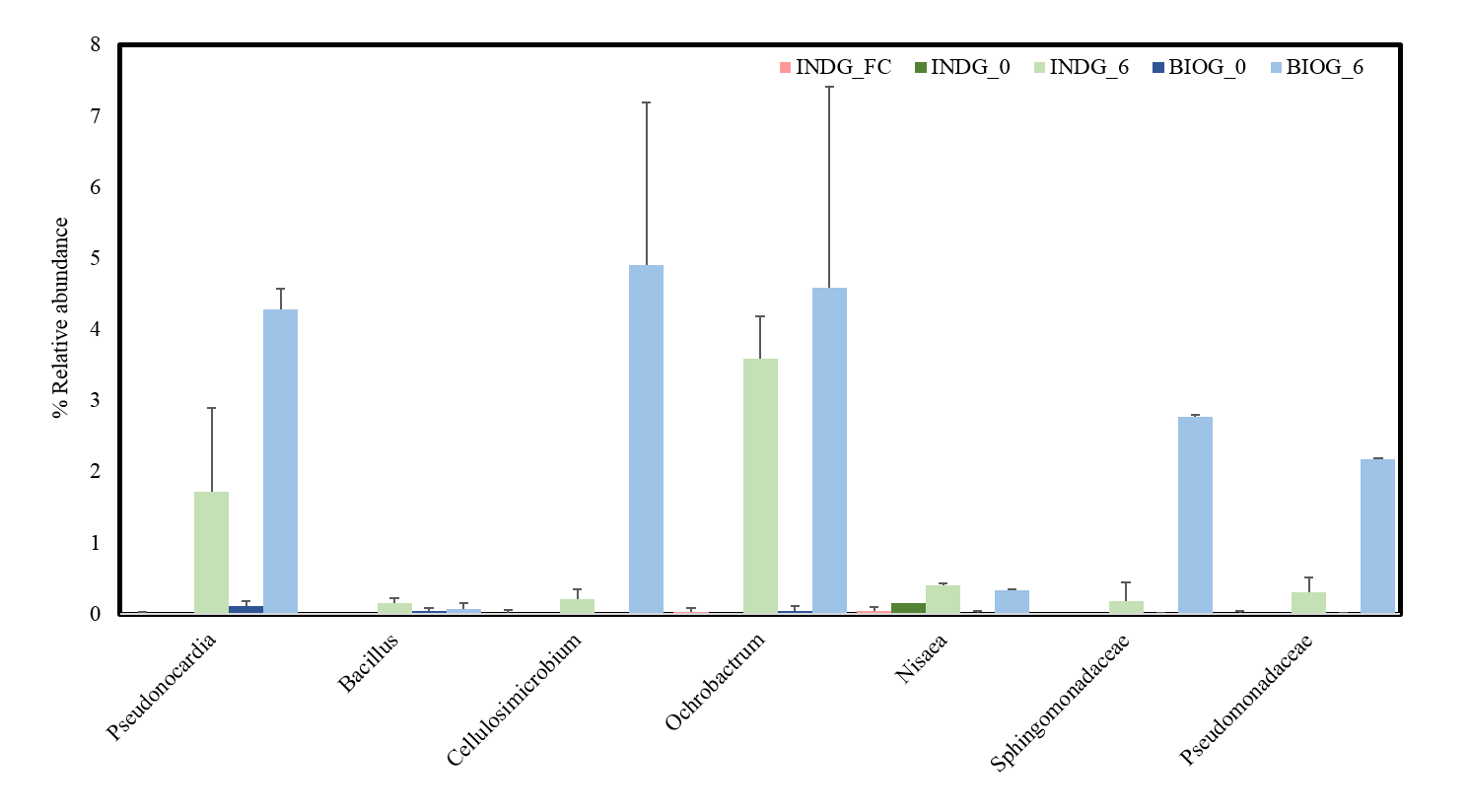

Supplement: S4 Fig — Abundances of biomarkers (enriched OTUs) in acclimated biofilm communities (INDG: Kruskal-Wallis and Wilcoxon rank-sum p<0.05 and LDA score >4 & BIOG: Kruskal-Wallis and Wilcoxon rank-sum p<0.05 and LDA score >3). (TIF) [file pone.0183984.s004.tif]
